# Effect of plant-derived exosome-like nanoparticles in ultraviolet-induced skin photoaging

**DOI:** 10.3389/fphar.2025.1721879

**Published:** 2025-12-04

**Authors:** Hai Dong, Yi Deng, Jiayan Li, Wen Lin, Yangyan Luo, Yuan Jiang

**Affiliations:** Clinical Medical College and The First Affiliated Hospital of Chengdu Medical College, Chengdu, China

**Keywords:** ultraviolet, photoaging, oxidative stress, inflammation, plant-derivedexosome-like nanovesicles

## Abstract

Long-term exposure to ultraviolet (UV) irradiation leads to skin photoaging. The mechanism of photoaging is complex, involving oxidative stress, inflammation, and collagen degradation. Certain active ingredients derived from plants possess strong antioxidant and anti-inflammatory properties and have been utilized in cosmeceuticals for cosmetic medicine. Plant-derived exosome-like nanovesicles (PELNs) contain bioactive constituents obtained from plant cells, which contribute to PELNs possessing multiple pharmacological functions, making them beneficial to skin care. Some researchers used PELNs as natural agents to attenuate UVB-induced skin photoaging, and their findings are critical for discovering novel anti-photoaging treatment modalities. In this mini-review, we focus on the related research of PELNs and UVB-induced skin photoaging, and introduce the protective role of PELNs. Finally, we will discuss the challenges and future directions in the development of PELNs for anti-photoaging.

## Introduction

Long-term exposure to sunlight can lead to significant skin damage, both in terms of aesthetic changes and structural degradation, which results in photoaging (Kaltchenko and Chien, 2025). The clinical features of photoaging include wrinkles, roughness, laxity, telangiectasia, and dyspigmentation (Ma et al., 2022; Gollogly et al., 2024). Besides creating an aged appearance, photoaging can also lead to various light-related skin conditions, such as melasma and actinic keratosis, and even increase the risk of skin tumors (Prawer, 1991; Kwon et al., 2019; Pfeifer, 2020). Ultraviolet (UV) radiation is the most prevalent component of solar radiation, accounting for 5%–10% of the solar radiation that reaches the Earth’s surface (Battie et al., 2014). However, UV is the most harmful external factor contributing to skin photoaging. UV radiation is categorized into three types: UVA (320–400 nm), UVB (280–320 nm), and UVC (100–280 nm). UVB radiation penetrates the epidermis, while UVA radiation can reach the dermis. Both of them can cause significant damage to skin structures and accelerate the process of photoaging. UVC radiation does not get to the Earth’s surface because of the effective absorption by the ozone layer (Gromkowska-Kępka et al., 2021; Salminen et al., 2022). Data indicate that over 83% of people aged 20 and above exhibit obvious signs of skin photoaging. Under 30 years old, 72% of men and 47% of women exhibit moderate to severe changes in skin texture (Zhou et al., 2025). Notably, the population that suffers occupational UVR exposure faces significant health risks, such as nonmelanoma skin cancer (NMSC). In 2019, 18,960 NMSC-related deaths were attributable to occupational UVR exposure. The global WHO/ILO Comparative Risk Assessment highlighted that occupational UV exposure is the third largest of the occupational carcinogens, only behind occupational exposures to asbestos and silica (Pega et al., 2023). Skin photoaging has become a public health concern. However, it can be largely preventable and reversible through effective prevention and treatment strategies. As early as the ancient Egyptian period, people used a mixture of rice bran, jasmine, and lupine to protect their skin from UV-induced damage. Modern research has demonstrated that secondary metabolites produced by plants, such as phenolic compounds, vitamin C, carotenoids, and other organic compounds, can serve as natural antioxidants, helping to protect the skin from photoaging (Petruk et al., 2018; Zhao et al., 2025). Certain active ingredients derived from these plants have been utilized in cosmeceuticals for cosmetic medicine due to their ability to reduce skin damage and promote skin repair by inhibiting inflammatory responses, reducing oxidative stress, enhancing DNA repair, and regulating melanin deposition (Chan et al., 2024). Existing evidence suggested that plant cells can secrete nanoscale vesicles with a double-layer membrane structure, which have a similar appearance and function to mammalian-derived exosomes (MDEs) (Mu et al., 2023). Interestingly, these plant-derived exosome-like nanovesicles (PELNs) contain bioactive constituents obtained from plant cells, including nucleic acids, proteins, lipids, and bioactive substances, which contribute to PELNs possessing multiple pharmacological functions, including anti-inflammatory, antioxidant, antiviral, and antitumor properties (Bai et al., 2024). Moreover, their stability, safety, biocompatibility, and specific tissue targeting contribute to their use as carriers for drug delivery (Kim et al., 2022a). In recent years, the therapeutic activities and application of PELNs have become a prominent research focus. Some researchers used PELNs as natural agents to attenuate UVB-induced skin photoaging, and their findings can provide new perspectives and stimulate further exploration of the effect of PELNs on human skin health. In this mini-review, we focus on the related research of PELNs and UVB-induced skin photoaging, and introduce the protective role of PELNs. Finally, we will discuss the challenges and future directions in the development of PELNs for anti-photoaging.

## Mechanism of skin photoaging

The mechanism of UV-induced skin aging involves multiple aspects: a) Oxidative stress: UV radiation can induce the production of ROS in skin cells, leading to oxidative stress. Oxidative stress not only damages cellular components such as lipids, proteins, and DNA, leading to cellular dysfunction, but also reduces the level of collagen in the extracellular matrix by promoting the expression of matrix metalloproteinases (MMPs), resulting in skin sagging and wrinkles. b) Inflammation: UV radiation can trigger the production of proinflammatory cytokines, resulting in inflammatory responses in the skin. Interleukin-1 alpha (IL-1α) and interleukin-1 beta (IL-1β) can upregulate the expression of MMPs, leading to a reduction in the level of collagen and elastin. Interleukin-6 (IL-6) cannot only induce the breakdown of collagen and elastin by increasing the expression of MMPs, but also can promote the formation of senescent cells, accelerating the aging process. c) Extracellular matrix degradation: UV radiation can induce the secretion of MMPs, leading to the breakdown of collagen and elastin. MMP-1 can break down intact fibrous collagen, and MMP-2, MMP-3, and MMP-9 can break down collagen fragments, leading to an increase in collagen protein breakdown in the dermis (Hajialiasgary Najafabadi et al., 2024). d) DNA damage: UVB radiation can directly or indirectly damage DNA, including the formation of pyrimidine dimers, oxidative base modifications, and DNA strand breaks. In addition, UVB radiation can induce telomere mutations, shortening, and telomerase dysfunction, facilitating cell death and photoaging (Zhao et al., 2025). e) Immunosuppression: UVB-induced immunosuppression can be attributed to the presence of suppressive immune cells, for instance, Treg cells. UVB radiation can trigger Treg cells to secrete IL-10, leading to the suppression of effector immune cells. UVB radiation can also elevate the expression and secretion of IL-10 and TGF-β in human keratinocytes. TGF-β can stimulate the expression of aryl hydrocarbon receptor (AhR), which consequently increases the differentiation of Treg cells. The accumulation of activated immunosuppressive cells in skin tissue can induce immunosenescence, promoting the process of photoaging (Mezrich et al., 2010; Salminen et al., 2022). f) Autophagy: As a self-protection mechanism of cells, autophagy can remove damaged proteins and organelles caused by UV radiation, and promote cell survival. However, repeated exposure to ultraviolet radiation can interfere with the autophagy process, leading to cellular dysfunction and the development of photoaging (Ma et al., 2022). g) Endoplasmic reticulum stress: UV radiation can activate the endoplasmic reticulum stress response within cells. Moderate endoplasmic reticulum stress has a protective effect on the skin, while excessive endoplasmic reticulum stress induces cell apoptosis and exacerbates skin aging (Tai et al., 2024). h) Other: Apoptosis, mitochondrial dysfunction, ferroptosis, skin adipose tissue collapse, and epigenetic changes, such as DNA methylation, histone modification, and dysregulation of non-coding RNA expression, are also involved in photoaging (Barnes et al., 2024; Teng et al., 2025; Yuan et al., 2025). Although the mechanism of photoaging is complex, oxidative stress and inflammation are key factors in the occurrence of photoaging.

## Biogenesis of PELNs

**FIGURE 1:**
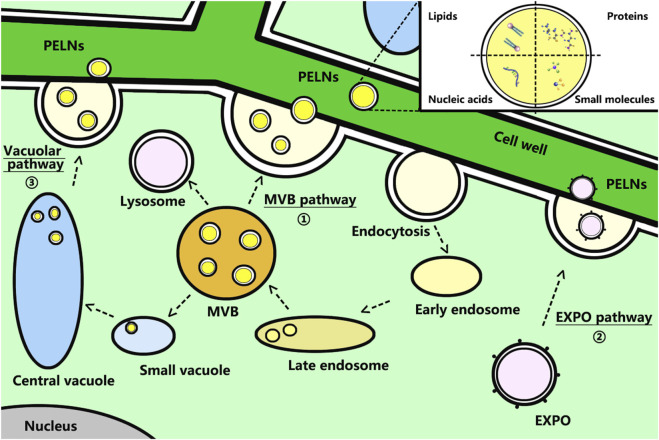
Schematic representation of the biogenesis and main components of PELNs. Route ① shows the MVB pathway: MVBs fuse with the plasma membrane to release PELNs. Route ② shows the EXPO pathway: EXPOs fuse with the plasma membrane to release PELNs. Route ③ shows the vacuolar pathway: vacuoles fuse with the plasma membrane to release PELNs. Abbreviations: PELNs, plant-derived exosome-like nanoparticles; MVBs, multivesicular bodies; EXPO, exocyst-positive organelle.

In recent years, researchers have extensively studied mammalian-derived exosomes (MDEs), especially their biogenesis. MDEs originate from the early endosomes formed by the invagination and endocytosis of the inner membrane. The early endosomes then mature into late endosomes, which contain bioactive content such as DNA, mRNA, miRNA, and proteins. Late endosomes bud off to form intraluminal vesicles (ILVs) and multivesicular bodies (MVBs). Subsequently, MVBs fused with the plasma membrane, resulting in their release into the extracellular environment as exosomes (Jiang et al., 2020; Wang et al., 2021; Krylova and Feng, 2023). The biogenesis of PELNs also involves a high resemblance to the MVB pathway with MDEs. In addition, researchers found two other pathways in the biogenesis of PELNs: the exocyst-positive organelle (EXPO) pathway and the vacuolar pathway (Zhao et al., 2024; Liu et al., 2025) (Figure 1). EXPOs are double-membrane organelles and morphologically similar to autophagosomes. EXPOs are distinct from MVBs and are not involved in the endocytic pathway. However, they can also fuse with the plasma membrane to release single-membrane vesicles known as exosomes (Wang et al., 2010). The vacuolar pathway involves vacuoles that fuse with the plasma membrane and release PELNs when the plant defends against fungal pathogens. The small vacuoles (SVs) derived from MVBs encapsulate cargo and fuse with the central vacuole, which then releases vacuoles, indicating potential connections between the vacuolar pathway and the MVB pathway (Cui et al., 2019). The cargo sorting mechanism of PELNs is still unclear. Scholars highlighted that the MVB pathway also requires the action of the endosomal sorting complex required for transport (ESCRT) complexes to actively and selectively incorporate RNAs, DNA, lipids, and proteins, similar to the biogenesis of MDEs. However, the composition profiles of ELNVs’ protein, lipid, and RNA content maintain significant discrepancies from those of MDEs. Notably, PELNs contain some bioactive substances from homologous plants, such as saponins, polyphenols, flavonoids, glucosinolates, terpenoids, and carotenoids, which contribute to PELNs playing corresponding bioactive roles after crossing various physiological barriers (Lian et al., 2022). Notably, their anti-inflammatory and antioxidant functions may be beneficial for skin photoaging. Based on the lipid fusion effect, PELNs can pass through the stratum corneum (SC) by the transcellular and intercellular routes, and they can even use the trans-follicle route to skin penetration (Dad et al., 2021). Given they can also carry drugs as carriers, PELNs can be envisaged as a novel way to anti-photoaging by transdermal drug delivery.

## PELNs and skin photoaging

Previous studies have demonstrated that PELNs have significant therapeutic potential in skin care, anti-aging, and injury repair. For example, coriander-derived exosome-like nanovesicles, grapefruit-derived extracellular vesicles, and wheat-derived nanovesicles effectively prevent oxidative stress, reduce inflammation, and promote wound healing in the skin (Şahin et al., 2019; Savcı et al., 2021; Wang T. et al., 2025). To identify relevant literature on PELNs and UVB-induced skin photoaging, we searched recent research from the past decade using electronic databases such as Science Direct, PubMed, and Web of Science. The articles found indicate that PELNs can also effectively attenuate the UVB-induced skin photoaging and produce positive results (Table 1).

**TABLE 1:** Literature examples of PELNs in anti-UV-induced photoaging.

PELNs source	Size (nm)	Zeta potential (mV)	Delivery method	Animal models	UV doses	Intervention cycle	Mechanism of action	References
*Lavender*	160.1	−26.6	—	ICR mice	First 3 days: 100 mJ/cm^2^ Every 2 days: Increased by 50 mJ/cm^2^ 14th day: 400 mJ/cm^2^	14 days	Downregulated the levels of IL-1β, IL-6, TNF-α, MMP-1, and MMP-3; upregulated the levels of COL-1	Li et al. (2025)
*Olea europaea*	50 to 500	−40.71 ± 0.28	HA/TA hydrogel	ICR mice	First 2 weeks: 1 MED Third week: 2 MED Fourth week:3 MED	4 weeks	Downregulated the levels of ROS, IL-6, SA-β-Gal, MMP-1, and MMP-3; upregulated the levels of SOD and COL-1	Wang et al. (2024)
*Aloe Vera Gel* and *Rind*	190 (gADNP); 160 (rADNPs)	−14.59 ± 0.68 (gADNP);-24.26 ± 0.84 (rADNPs)	Microneedle	ICR mice	First week: 1 MED Second week: 1.5 MED Third week: 2 MED Fourth week:2 MED	4 weeks	Downregulated the levels of P53, P21, CXCL1, IL-6,TFG-β, SA-β-gal, MDA, and MMP-1; upregulated Nrf2/ARE pathway	Sun et al. (2025)
Grape	237.2 ± 0.52	−35.96 ± 10.88	—	Kunming mice	First 2 weeks: 1 MED Third week: 2 MED Fourth week: 3 MED Fifth to eighth week: 4 MED	8 weeks	Downregulated the levels of IL-6,IL- 1β, MMP-1, p53, p21, ROS, SA-β-gal, and MDA; upregulated the levels of SOD	Wang et al. (2025a)

PELNs, Plant-derived exosome-like nanovesicles; UV, ultraviolet; ADNP, aloe-derived exosome-like nanoparticles; HA, hyaluronic acid; TA, tannic acid; ICR, institute of cancer research; MED, minimal erythema dose; COL-1, Collagen 1; ROS, reactive oxygen species; SA-β-Gal, Senescence-associated β-galactosidase; SOD, superoxide dismutase; MDA, malondialdehyde.

## Lavender exosome-like nanoparticles (LELNs)

*Lavender* (*Lavandula angustifolia*) extracts are commonly used as skincare products to treat UV-induced skin damage by effectively alleviating oxidative stress caused by UV exposure, promoting collagen synthesis, and delaying skin aging. Li and colleagues have isolated *lavender* exosome-like nanoparticles (LELNs) and evaluated their protective effects against UVB-induced skin photoaging *in vitro* and *vivo*. They first isolated LELNs from dried lavender flowers of the “Space Blue” variety using ultracentrifugation and sucrose density gradient centrifugation, and they characterized the particle size, zeta potential, and morphology of the LELNs. LELNs are characteristic cup-shaped vesicles with intact membrane structures, and their average vesicle diameter and zeta potential were 160.1 nm and −26.6 mV, respectively. In HaCaT cells, LELNs can protect them from UVB-induced damage and inflammatory response and increase cell viability and collagen content. After treating with LELNs before each time of UVB exposure for 14 days, the results of histopathological and biochemical analyses showed that the skin of the LELNs-treated mice with back hair removal remained relatively smooth with only mild desquamation, and the epidermal thickness and collagen degradation of the dorsal skin significantly decreased compared to the photoaging models. The researchers then found that the specific miRNAs in LELNs, such as cpa-miR166e and zma-miR166h-3p, play critical roles in mitigating UVB-induced skin photoaging through multiple pathways, including DNA repair and replication pathways, oxidative stress response mechanisms, inflammation pathways, and collagen synthesis pathways. This study provides strong evidence for LELNs as a therapeutic strategy for preventing and treating UVB-induced skin photoaging (Li et al., 2025).

## Oleaeuropaea leaf exosome-like nanovesicles (OLELNVs)

*Olea europaea* leaf extract (OLEX) is rich in antioxidant phenolic compounds such as oleuropein and hydroxytyrosol, which contribute to its significant antioxidant, anti-inflammatory, and anti-aging properties. However, its low transdermal efficiency and cytotoxicity will impede its application in skincare. Wang and colleagues isolated the *oleaeuropaea* leaf exosome-like nanovesicles (OLELNVs) from *O. europaea* leaves, which were from local olive farms. They observed that OLELNVs have a circular shape with a distinct exosome-like lipid bilayer membrane structure, and their size ranges from 50 to 500 nm, and the zeta potential was −40.71 ± 0.28 mV. In contrast to OLEX, OLELNVs exhibit a greater cell compatibility, free radical scavenging capacity, superior transdermal capabilities, and no cytotoxicity. Utilizing the UV absorption capacity of the hyaluronic acid (HA)/tannic acid (TA) hydrogel system, Wang et al. incorporated OLELNVs into the HA/TA hydrogel to form the OLELNVs@HA/TA hydrogel system, which can integrate the anti-UV radiation and protect the skin from photoaging. ICR mice with hair removed from the back were treated with OLELNVs@HA/TA before and after each UV irradiation, every other day for 4 weeks. They found that OLELNVs@HA/TA protected ICR mice from sunburn, attenuated the degradation of skin collagen and elastic fibers by UV irradiation, reduced cellular senescence and apoptosis induced by UV radiation, and enhanced the skin’s antioxidant defenses, preserving skin structure and function. Notably, the HA/TA hydrogel enhanced the stability of OLELNVs and sustained release of OLELNVs in skin tissue, suggesting that hydrogels may be an ideal carrier of PELNs in skincare (Wang et al., 2024).

## Aloe-derived exosome-like nanoparticles (ADNPs)

*Aloe vera* has been widely used in both food and herbal medicine due to its leaves and roots containing a variety of pharmacologically active ingredients, including aloe-emodin, aloin, β-sitosterol, quercetin, flavonoids, anthraquinones, and phenolic compounds (Sánchez et al., 2020; Kaur and Bains, 2024). Notably, aloe vera and its active ingredients are highly beneficial for protecting against UV damage and delaying skin aging, making it a valuable medicinal plant for skincare (Liang et al., 2021; Michalak, 2023). Previous studies have confirmed that *aloe*-derived exosome-like nanoparticles (ADNPs) exhibit anti-inflammatory, antioxidant, and prompt wound-healing properties (Kim et al., 2021; Kim and Park, 2022b). Sun and colleagues investigated the potential of ADNPs in delaying UV-induced skin aging. They isolated gADNPs and rADNPs from *aloe vera gel* and *aloe vera rind*, respectively. The gADNPs and rADNPs are negatively charged nano-sized particles with a typical oval or cup-shaped phospholipid bilayer structural morphology. They observed that gADNPs and rADNPs can promote migration and proliferation of UVA-induced photoaging cell models. In addition, these particles can scavenge ROS and reduce the expression of the DNA damage markers (γ-H2AX, 53BP1) and aging marker β-gal in UV-exposed cells. These findings suggested that ADNPs can protect skin cells from UV damage and alleviate photoaging. Then the researchers used a microneedle roller to deliver gADNPs and rADNPs to the dermis of the dorsal skin of ICR mice, once a week for 4 weeks. They found that this intervention can protect mice against UV-induced skin photoaging via activating the Nrf2/ARE pathway. This study provides substantial evidence of ADNPs’ anti-skin photoaging properties *in vitro* and *vivo* (Sun et al., 2025).

## Grape-derived exosome-like nanoparticles (GENs)

Grapes are a fruit that is rich in a variety of antioxidants. Their natural ingredients, such as resveratrol and polyphenols, can reduce oxidative stress and promote cellular repair and metabolism, possessing anti-aging effects for the skin (Nassiri-Asl and Hosseinzadeh, 2009; Ndiaye et al., 2011). However, the skin barrier limits the absorption of these beneficial ingredients, and using higher concentrations may actually harm skin tissue. To overcome these limitations, Wang and colleagues utilized grape-derived exosome-like nanoparticles (GENs) to protect the skin from the damaging effects of UV exposure and photoaging and investigated their molecular mechanisms. They first characterized the size, zeta potential, and morphology of GENs from Kyoho grapes (China). The results showed that the GENs have distinctive features of an exosome-like lipid bilayer structure, with an average diameter of 237.2 ± 0.52 nm, and their zeta potential was −35.96 ± 10.88 mV. In HaCaT Cells exposed to UV, GENs can improve the photoaging of cells by their regulatory effects on cell proliferation and differentiation. In the mouse photoaging model, injecting GEN into the back skin before UV irradiation can improve UV-induced photoaging by reducing epidermal thickness, minimizing collagen fiber loss in the skin, and enhancing the skin’s antioxidant capacity. Therefore, the researchers suggested that GENs hold potential as an innovative approach for combating photoaging and improving skin health (Wang M. et al., 2025).

## Summary and future perspective

**FIGURE 2:**
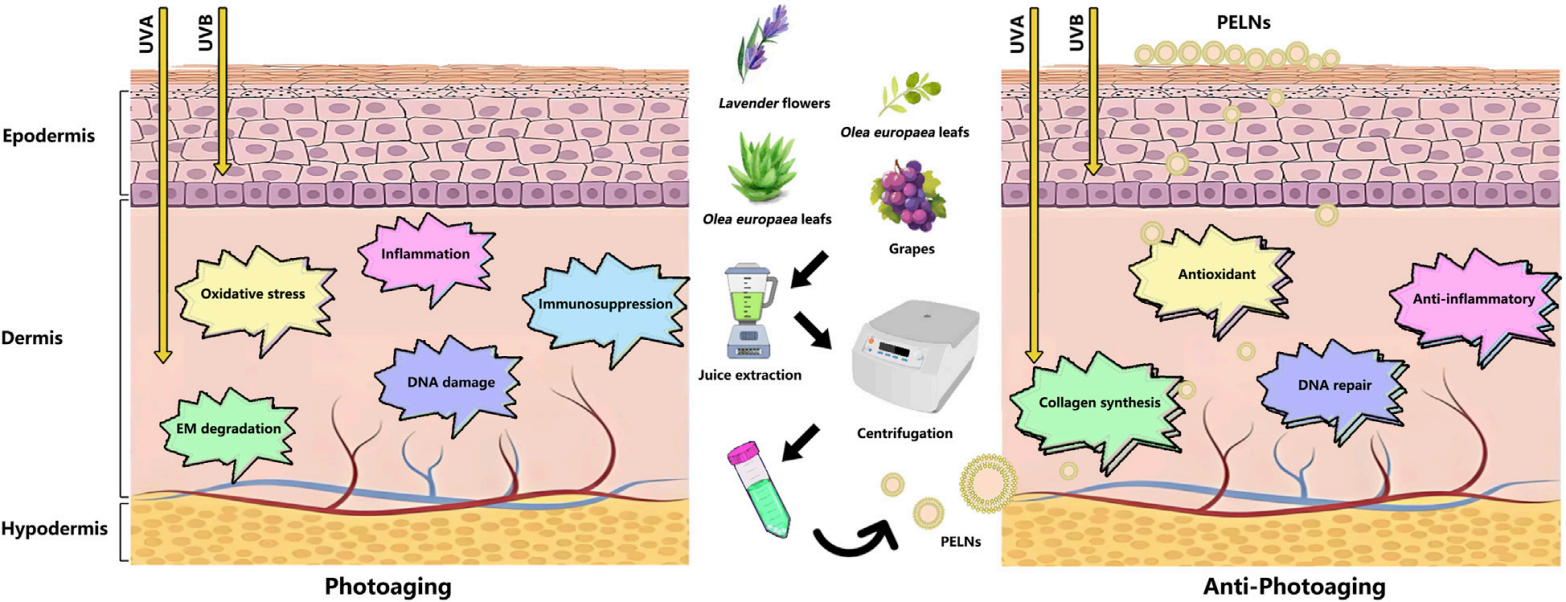
Schematic representation of the mechanism of ultraviolet-induced skin photoaging and the protective effect of PELNs. Abbreviations: PELNs, plant-derived exosome-like nanoparticles; EM, extracellular matrix; UVA, ultraviolet A; UVB, ultraviolet B.

Compared to current commercialized sunscreen products and medication treatments, PELNs have some advantages in anti-photoaging. First, PELNs from plants with non-toxicity and non-immunogenicity contribute to their non-irritating nature to the skin. Second, PELNs with a good biocompatible nature can penetrate the stratum corneum and fuse with skin lipids, enhancing their diffusion and absorption. Third, PELNs have rich bioactive compounds, which contribute to their anti-inflammatory, antioxidant, DNA repair, and collagen synthesis properties. These properties also align with the pathogenesis of photoaging (Figure 2). Fourth, PELNs can not only transfer their bioactive compounds to specific target cells by crossing biological barriers but also act as natural drug carriers, transporting drugs and exerting the therapeutic effects in the body. However, the research on PELNs in anti-photoaging remains in its early stages, and our understanding of their function is limited. It is unclear how PELNs interact with skin cells and how skin cells uptake PELNs, which may affect the absorption, distribution, excretion, and metabolism of PELNs in the skin tissue. PELNs contain a variety of bioactive substances that may cause unknown side effects to the skin tissue. For instance, special bioactive substances may cause a photosensitive effect, damaging skin tissue under UV exposure. The plants from different areas and harvest seasons that obtained PELNs will differ in the content of bioactive substances.

Researching of PELNs will also encounter the same challenges as MDEs. There is a lack of unified standards for the separation and extraction of PELN, and related regulatory procedures are absent. The varying yields and purities of PELNs by different methods will cause heterogeneity and inconsistent results. The suitable delivery strategy of PELNs is also a key consideration, such as the application of hydrogels or microneedle patches, which can enhance their stability and improve dermal penetration in skin tissue. The above issues will limit the clinical application of PELNs in anti-photoaging. Of course, concerns about the stability, reproducibility, and large-scale manufacturing of PELNs could impact their transition into clinical skincare products in the future. However, we are optimistic that researchers will resolve these issues, as has been the case in any newly emerging field. PENs demonstrate a promising therapeutic potential that may benefit patients with UV-induced photoaging, deserving further investigation in the future.
